# Design and application of personalized exercise prescription for primary osteoporosis

**DOI:** 10.1097/MD.0000000000032857

**Published:** 2023-02-17

**Authors:** Yongjie Li, Dakuan Zhang, Shenyu Fu, Mengling Liu, Hongju Liu

**Affiliations:** a Department of Rehabilitation Medicine, Guizhou Provincial Orthopedics Hospital, Guiyang, China.

**Keywords:** bone mineral density, exercise prescription, primary osteoporosis, protocol, randomized controlled trial

## Abstract

Regular exercise has been shown to have a beneficial effect on primary osteoporosis (POP). However, current exercise prescriptions have limitations such as insufficient individualized features and low participant compliance, which in turn limit their application in clinical practice. In this study, we propose to establish a personalized exercise prescription based on the Chinese traditional exercise—Yi Jin Jing, combined with treadmill exercises and strength training, and then observe its effects on pain, muscle strength, balance, bone mineral density (BMD) and bone metabolic indexes in patients with POP.

**Methods:**

This trial is a single-center, randomized, assessor-blinded, controlled clinical study. We will recruit 40 patients with POP and assign them to the control group and the experimental group in a 1:1 ratio according to the intended protocol. The control group received 24 weeks of conventional medication and health promotion, and the experimental group received 24 weeks of personalized exercise prescription intervention (Yijinjing, treadmill exercises, and strength training). Outcome measures include pain (visual analogue scale), muscle strength (bilateral upper limb grip strength and isometric muscle strength of the trunk, hip, and knee), balance (Balance test with eyes open and closed while standing on one leg), and BMD (Lumbar spine 2–4 and femoral neck). In addition, bone metabolism indicators include parathyroid hormone, osteocalcin, 25-hydroxyvitamin D3 (1,25(OH) 2D3), type I procollagen aminoterminal peptide and type I collagen carboxy-terminal peptide. Outcome measures will be assessed before and after 24 weeks of intervention. Statistical analysis was performed by SPSS22.0.

**Discussion:**

This trial aimed to enrich the content and form of exercise rehabilitation prescriptions for patients with POP, which is conducive to improving the exercise rehabilitation effect and quality of life in this population.

## 1. Introduction

Primary osteoporosis (POP) in clinical practice refers to a systemic bone disease typically characterized by reduced bone mass and microstructural damage to bone tissue, mostly in middle-aged and elderly people.^[1]^ Its symptoms include low back pain, shortening of body length, fracture, and decreased respiratory function, which seriously reduces the quality of survival of patients.^[2]^ Currently, pharmacological interventions are still the most common treatment for POP, however, long-term drug use is costly and has the potential risk of increasing the occurrence of various adverse effects.^[3]^ Therefore, it is crucial to explore safer and more efficient alternative therapies. Previous studies have shown that regular exercise can effectively enhance BMD and reduce the incidence of osteoporosis (OP) in middle-aged and elderly people with a high safety profile.^[4,5]^ The development of an accurate and appropriate exercise prescription has been of great interest to medical staff and patients as a relatively economical, efficient, and safe treatment method in the clinical treatment of POP.^[6]^

As a specific traditional sport in China, traditional Chinese health-preservation exercises are rich in form and easy to accept the load, making them more suitable for middle-aged and elderly people to participate.^[7]^ In our previous study, we found that Yi Jin Jing was optimal in improving pain, bone density, and bone formation in POP patients compared with TaiChi, Ba Duan Jin, and Wu qin xi through a network Meta-analysis.^[8]^ Therefore, Yi Jin Jing may be preferred in clinical practice. In addition, treadmill exercises and strength training have been shown in the literature to have significant effects on the improvement of BMD and bone metabolism.^[9,10]^ However, previous exercise protocols have some limitations. First, most of the studies were very vague in setting and monitoring the exercise intensity, and failed to make individualized exercise intensity programs according to the patient’s physical condition, which may lead to the intervention effect not achieving the expected effect and even causing secondary injuries. Second, the single and poorly targeted intervention means are more prominent, which will directly lead to lower patient compliance and difficulty in completing rehabilitation training. Finally, current assessments of POP focus on changes in bone mineral density (BMD), ignoring factors such as muscle strength and physical function. Previous studies have also shown that the decline of the skeletal muscular system in patients with POP causes muscle atrophy and bone loss, which in turn causes loss of muscle strength, decreased motor ability and balance, and increases the risk of falls, ultimately leading to a reduced quality of life.^[11,12]^

Therefore, this trial proposes to establish a personalized exercise prescription based on Yi Jin Jing and combined with treadmill exercises and strength training, and then observe its effects on pain, muscle strength, balance, bone density, and bone metabolism indexes in POP patients. The aim of this study is to enrich the content and form of exercise rehabilitation prescriptions for POP patients, to improve the effect of exercise rehabilitation in this population, and to improve the quality of life.

## 2. Methods

### 2.1. Study design

**Figure 1.**
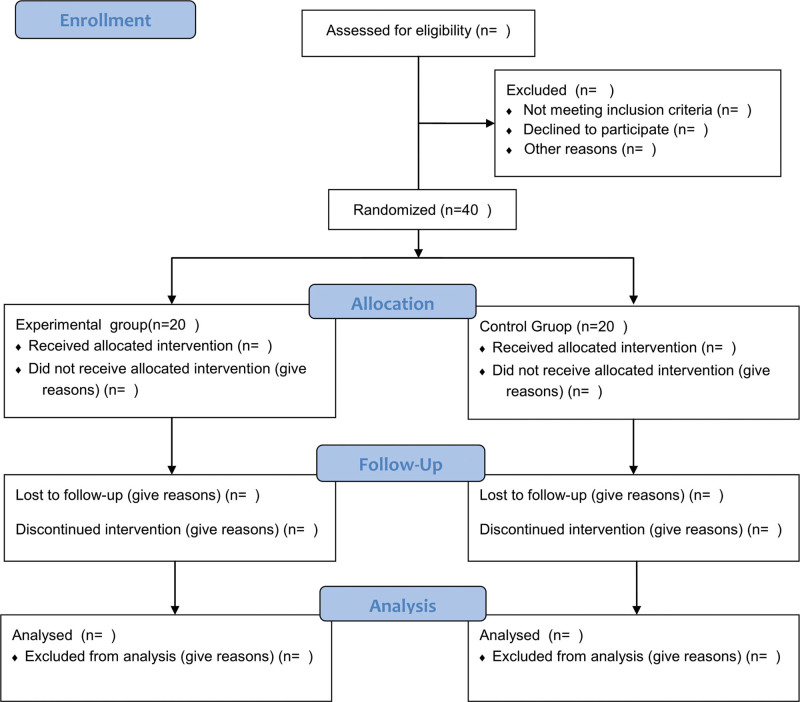
Flow chart of the study.

**Figure 2.**
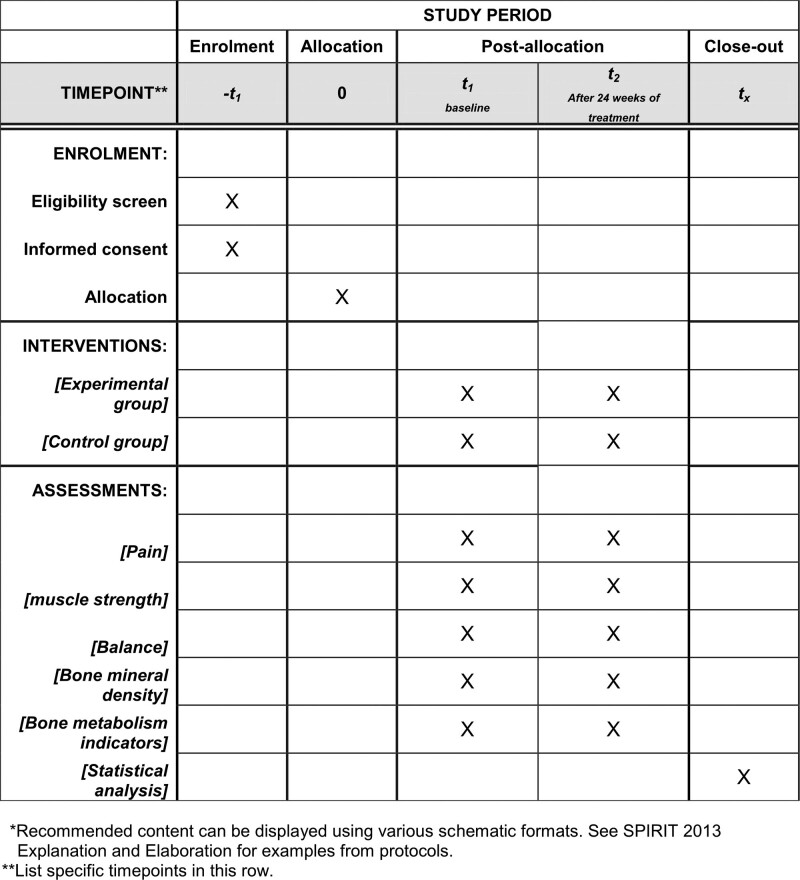
SPIRIT figure as recommended by 2013 SPIRIT statement.

This trial is a single-center, randomized, assessor-blinded, controlled study. We will recruit 40 POP patients and assign them to the control group (conventional medication and health education) and the experimental group (personalized exercise prescription based on Yijinjing) in a 1:1 ratio according to the intended protocol. Before randomization, all patients were required to sign an informed consent form. The flow chart of the study is shown in Figure 1. the study protocol is based on the SPIRIT reporting guidelines, where the SPIRIT figure (schedule of enrollment, interventions, and assessments) is shown in Figure 2, and the checklist is shown in Supplementary Material 1 (http://links.lww.com/MD/I526). The study started in November 2022 at Guizhou Provincial Orthopedics Hospital and is expected to end in October 2023.

### 2.2. Clinical registration and ethical approval

This study complied with the Declaration of Helsinki and was approved by the Ethics Committee of Guizhou Provincial Orthopedics Hospital (approval number: LW2021101401). In addition, this study was also registered with the Chinese Clinical Trials Registry under the registration number ChiCTR2200056780.

### 2.3. Inclusion criteria

1. Included patients will meet the diagnostic criteria of the guidelines for the diagnosis of POP (2017).^[13]^
2. Bone density test T value ≤ −2.5 and moderate pain in the low back (3 < visual analogue scale score < 7).
3. Age between 50 and 75 years old, male and female gender was not limited.
4. Signed informed consent and good compliance.

### 2.4. Exclusion criteria

1. Secondary OP and other causes of OP.
2. Combined bone diseases such as bone tuberculosis and malignant bone tumor.
3. Severe abnormalities in cardiovascular, liver, kidney, stomach, and other system functions.
4. Persons with cognitive dysfunction or mental illness.
5. Those who have received medication and regular exercise in the past 3 months.
6. Those who cannot tolerate exercise.

### 2.5. Sample size estimation

Sample size estimation was performed by G.power3.1. Statistical efficacy analysis was performed based on a previous study.^[14]^ A two-sided test was selected, α was set to 0.05, Statistical power was set to 0.8, and the sample size estimation resulted in 34 participants. Considering a 20% dropout rate, the finalized sample size was 40 individuals.

### 2.6. Randomization, concealment, and blinding

A random sequence will be generated by SPSS 22.0, and patients will be randomly assigned to experimental and control groups based on a 1:1 ratio. The randomization grouping results will be stored in opaque, sealed envelopes with serial numbers marked on the cover. These envelopes were opened only before the official start of the intervention.

Blinding of therapists and patients is not possible due to the limitations of the intervention method. Data collection was performed and recorded by specialized personnel, and data analysis was performed by an independent statistician. The grouping of patients was not known for the above population.

### 2.7. Intervention method

The control group will be given conventional medication and health education. The patients will be advised to take a calcium supplement, Calcium Carbonate and Vitamin D3 Granules (Beijing, China), which contains 500 mg of calcium and 200 IU of vitamin D in one sachet twice a day for 24 weeks. Health education includes informing patients about the dangers of OP, maintaining a good lifestyle and diet, and taking their medication on time.

#### 2.7.1. Experimental group.

In addition to conventional medication and health education, the experimental group will be given Yi Jin Jing exercise, treadmill exercises and strength training. Patients will wear Polar heart rate belt during training, and the rehabilitation therapist will monitor the patients’ exercise in real-time through the heart rate bands and make an immediate assessment of the patient’s fatigue level according to the Borg scale. Patients should stop the training immediately if they showed chest tightness, shortness of breath or vertigo during the training. All patients should try to maintain their pre-experimental daily routine and dietary habits and be prohibited from participating in other regular exercises. The patient’s daily activities, diet and, rest habits will be understood through regular questionnaires and telephone interviews.

(1) Yijinjing exercise protocol

The patients will be taught by a dedicated rehabilitation therapist for 24 weeks, with a learning period for the first 4 weeks and an intensive training period for the last 20 weeks, practicing 3 times a week (Mondays, Wednesdays and Fridays) for 30 minutes each time, with a 5 minutes warm-up and relaxation.

Yijinjing exercise consists of 12 movements. Its intensity is quantified by the target heart rate, which is monitored in real-time by the patient wearing a Polar heart rate belt. The target heart rate is based on a reserve heart rate of 60% to 70% for moderate intensity aerobic training. The specific algorithm for the target heart rate is^[15]^: target heart rate = reserve heart rate *(60% to 70%) + resting heart rate, reserve heart rate = 220 - age - resting heart rate.

(2) Treadmill exercises protocol

The intensity of treadmill exercises is still monitored by the target heart rate. Patients will wear a Polar heart rate belt and first performs an acclimatization run on the treadmill, followed by a formal run. The running speed is gradually increased, but the heart rate is generally controlled within the range of the target heart rate. If the patient’s heart rate is outside or below that range, the intensity of the exercise is reduced or increased as appropriate. The running time is 30 minutes, including a 5-minutes warm-up. Exercise frequency is 3 times/week (Monday, Wednesday, and Friday) for 24 weeks.

(3) Strength training protocol

**Figure 3.**
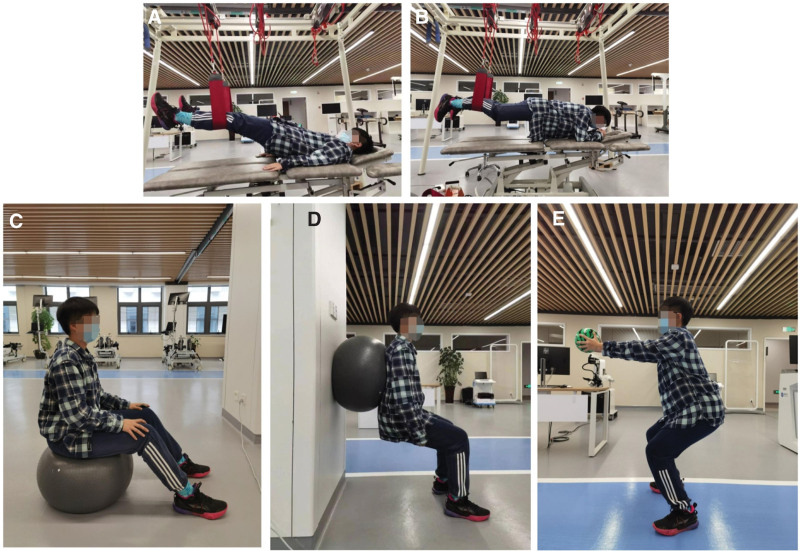
Strength training protocol. (A) Bilateral bridge. (B) Plank. (C) Seated training. (D) Swiss ball wall squats. (E) Ball squats.

The strength training consists of five movements: bridge exercise, plank, seated training, swiss ball wall squats and ball squats, see Figure 3A to E. Each movement will be repeated 10 to 15 times and practiced 3 times a week (Monday, Wednesday, and Friday) for 30 minutes each time for a total of 24 weeks. The training should be easy first and then difficult, and the exercise load should be controlled at the target heart rate, so that patients feel fatigue and slight muscle soreness. Patients will be instructed not to hold their breath with force and to pay attention to safety.

(1) Bilateral bridge

The bridge exercise is based on an unstable support surface. The patients are placed in the supine position, and the proximal ends of both lower legs are placed in the wide sling, and the trunk, knee, shoulder, and hip joints are maintained in the same line by raising the hips and extending the hips, and the movement lasts for 10 seconds.

(2) Plank

The patients are placed in a prone position, with both elbows bent over the bed, and the proximal ends of both lower legs set into the wide sling, then the body leaves the bed, ensuring that the ankle, hip, shoulder and head remain in a straight line. the movement lasts for 10 seconds.

(3) Seated training

The patients sit on the swiss ball with the trunk neutral and both lower extremities naturally separated. The therapist instructs the patient’s pelvis to tilt and rotate anteriorly, posteriorly, left and right on the swiss ball, maintaining each position for 5 seconds.

(4) Swiss ball wall squats

The patients squat with the back against the Swiss ball and holds the movement for 15 seconds, followed by standing up. Each movement is repeated 10 to 15 times depending on the situation.

(5) Ball squats

The patients maintain a deep squat, holds the upper limb straight and lifts the gravity ball (2 kg) 15~20 times, paying attention to the sunken shoulder and squatting posture.

### 2.8. Outcome measures

The patients were tested for outcome measures before and at the end of treatment.

(1) Pain

The patient’s low back pain level is assessed by the visual analogue scale.^[16]^ The score ranges from 0 to 10, with higher scores indicating greater pain.

(2) Muscle strength

Muscle strength is evaluated by grip strength of both upper limbs and isometric muscle strength of the trunk, hip and knee joints. Grip strength is assessed by an electronic grip strength meter. Isometric strength of the trunk and knee is evaluated by the Biodex system with a centripetal contraction mode. The test speeds are slow contraction (60°/s) and fast contraction (180°/s), and the test types are flexion and extension. The test sites were the left and right sides of the body, with no right or left side of the trunk. The outcomes include peak torque, average peak torque, average power, and peak flexor-to-extensor torque ratio.

(3) Balance

The static balance test will be conducted by the Italian Tecnobody balance system. Patients will stand with their right foot in the center of the pressure platform, then bend their left knee backward by 90° while crossing their hands at the waist, and tried to maintain standing with eyes open and eyes closed for 30 seconds each, with 15 seconds rest between groups. Static balance outcomes include the distance of plantar center of gravity pressure movement (mm), the area of movement trajectory (mm^2^), the deviation of the center of gravity in the X-axis and Y-axis (mm), and the average movement speed in the anterior-posterior and left-right directions (mm/s).

(4) Bone mineral density

BMD will be measured by Dual-energy X-ray absorptiometry at lumbar spine 2-4(L_2-4_) and femoral neck.

(5) Bone metabolism

After fasting for more than 12 hours, 10 mL of blood will be drawn from elbow vein in the morning of the next day, and serum bone metabolism indexes will be measured using a fully automated enzyme-labeled immunoassay instrument. The serum bone metabolism indexes include parathyroid hormone, osteocalcin, 25-hydroxyvitamin D3 (1,25(OH) 2D3), type I procollagen aminoterminal peptide and type I collagen carboxy-terminal peptide.

### 2.9. Statistical analysis

Statistical analysis will be performed by SPSS 22.0. If the data conforms to the normal distribution, paired *t* test is used for intra-group comparison and an independent-sample *t* test for inter-group comparison; a non-parametric test is conducted when the data does not conform to normal distribution. Differences are considered statistically significant at *P* < .05.

### 2.10. Data management and monitoring

Electronic data collected will be stored in encrypted spreadsheets, and paper data will be kept in a file cabinet and guarded. In addition, the patient’s signed informed consent form and the original case notes will be stored in the file cabinet. A data monitoring committee, independent of the investigator, will be established to review the quality and integrity of the data at regular intervals (1 month).

## 3. Discussion

With the increasing trend of population aging, the elderly are vulnerable to POP and has become a global public health problem.^[17]^ Among them, the incidence of osteoporotic fractures exceeds 40%, which can lead to pain, depression, loss of functional independence, decreased quality of life, and increased economic burden and mortality after fracture.^[18]^ In recent years, the ability of exercise to prevent the occurrence of POP in the elderly has been confirmed by scholars.^[19]^ However, most of the exercise prescriptions developed in previous studies have certain limitations: they are not personalized and quantified, which may not only lead to failure to achieve the expected efficacy, but also may even cause secondary injury; the interventions are in a single form, which may easily lead to poor patient compliance. Therefore, we developed a personalized exercise prescription for patients with POP. The program is based on the traditional Chinese exercise -Yi Jin Jing, and combines aerobic exercise and strength training to make patients actively complete exercise training through multi-modal exercise types. In order to ensure the exercise intensity of the patient, we equipped the patient with a Polar heart rate belt to enable the patient to complete the exercise program within the target heart rate range.

In addition, previous studies have tended to focus on skeletal health, while other physical functions of patients with POP have been neglected. Therefore, in order to systematically evaluate the efficacy of the exercise prescription of this protocol in patients with POP, we not only measured BMD and serum bone metabolism indexes but also observed changes in pain, muscle strength and balance. A comprehensive evaluation can effectively demonstrate the efficacy of this program and provide an evidence-based basis for clinicians to select an exercise program.

This study also has some limitations. First, due to the intervention modality, this study could not be blinded to therapists and patients. Second, due to sample size and single-center limitations, the results of this study may have potential bias.

Overall, this study will optimize a new personalized exercise prescription for improving POP and provide new insights for clinical practice.

## Author contributions

**Methodology:** Hongju Liu.

**Writing – original draft:** Yongjie Li, Dakuan Zhang.

**Writing – review & editing:** Shenyu Fu, Mengling Liu.

## Supplementary Material
